# Regional Cerebral Blood Flow in Mild Cognitive Impairment and Alzheimer's Disease Measured with Arterial Spin Labeling Magnetic Resonance Imaging

**DOI:** 10.1155/2017/5479597

**Published:** 2017-03-01

**Authors:** Alba Sierra-Marcos

**Affiliations:** ^1^Department of Neurology, Barcelona Medicine and Surgery Institute (IMECBA), Barcelona, Spain; ^2^Alzheimer Disease Research Unit, CIEN Foundation, Queen Sofia Foundation Alzheimer Center, Madrid, Spain

## Abstract

Alzheimer's disease (AD) depicts dynamic changes in regional brain function from early stages of the disease. Arterial spin labeling- (ASL-) based MRI methods have been applied for detecting regional cerebral blood flow (rCBF) perfusion changes in patients with AD and mild cognitive impairment (MCI). Nevertheless, the results obtained from ASL studies in AD and MCI are still controversial, since rCBF maps may show both hypoperfusion or hyperperfusion areas in brain structures involved in different cognitive functions. The goal of this review is to provide the current state of the art regarding the role of ASL for detecting distinctive perfusion patterns in subjects with MCI and/or AD. The ability to obtain this information using a noninvasive and widely available modality such as ASL should greatly enhance the knowledge into the broad range of hemodynamically related changes taking place during the cognitive decline process in AD.

## 1. Introduction

Alzheimer**'**s disease (AD) is a devastating neurodegenerative disorder with an important socioeconomic impact [1]. Mild cognitive impairment (MCI) is considered the prodromal phase of AD, and it is characterized by early cognitive symptoms not severe enough to meet the criteria for dementia [2]. With the prospect of disease-modifying therapies, it is desirable to detect signs of neurodegeneration at an earlier stage of the disease, before neuronal cell destruction is detectable at structural MR imaging as atrophy. Changes in regional brain function may be more dynamic and provide even greater sensitivity to early disease, disease progression, or responses to therapy than changes in regional gray matter volume depicted by structural MRI [3, 4].

Arterial spin labeling- (ASL-) based MRI methods have been applied for detecting regional cerebral blood flow (CBF) perfusion changes in patients with AD or MCI [5–16]. Since the inception of this technique more than 20 years ago [17], the quality of ASL-derived perfusion maps has reached a level that makes the method useful for many clinical and research applications. Important advantages of this technique are its noninvasiveness (since magnetically labeled protons in blood are used as a tracer) and short acquisition time at high magnetic field strengths (3.0 T) [18]. ASL has been validated extensively against other methods that use exogenous contrast agents, such as Positron Emission Tomography (PET) [19], and ASL implementation are now commercially available on all major MRI platforms, with demonstrated reproducibility in multicenter studies [20, 21]. The ability to quickly and noninvasively obtain CBF maps should greatly enhance the understanding of the hemodynamic mechanisms related to neurodegeneration [17, 22]. Since MRI scanners are more widely available than PET scanners (and also less expensive), ASL might become an alternative for FDG-PET [23, 24], allowing a higher throughput of patients. However, an important drawback for the implementation of ASL in clinical settings has been the lack of standardized protocols. Recently, international guidelines [25] and consensual initiatives have been developed to account for this issue.

Nevertheless, the results obtained from ASL studies in AD and MCI are still controversial. The goal of this review is to provide the current state of the art regarding the role of ASL for detecting distinctive perfusion patterns in subjects with MCI and/or AD.

## 2. A Brief Overview of the ASL Technique

ASL technique was developed in the 90s, using electromagnetically labeled arterial water as a standard diffusible tracer method for measuring perfusion. Proximal ASL alters the total magnetization of the protons in the arterial water of the distal tissue, and the extent of this alteration is determined by comparison of labeling images with control images. With an additional knowledge of the regional T1 of tissue, quantitative blood flow maps can be generated, since the electromagnetically labeled tracer has a decay rate of T1, allowing perfusion of the tissue and microvasculature to be detected [26].

The existing ASL techniques can be sorted out in two categories: “continuous (CASL)” or “pseudocontinuous (PCASL)” and “pulsed (PASL)” techniques, depending on the spatial extent and the duration of the spin labeling. In CASL/PCASL, labeling occurs as blood flows through a single labeling plane over a long period (1–3 s), whereas in PASL a slab of tissue, including arterial blood flow, is labeled using a single short radiofrequency pulse or limited number of them, with a total duration of 10–20 ms. The labeling efficiency and the ease of the incorporation into a clinical setting are superior with PASL, whereas the SNR is superior with CASL.

## 3. An Overview of the Literature in ASL and MCI/Dementia

Current knowledge regarding ASL hypo/hyperperfusion patterns in AD is still controversial. An important drawback of available studies is the heterogeneity of participant**'**s demographics, clinical diagnosis (involving cognitively healthy elderly population, different types of MCI, AD, other dementia types, etc.), neuropsychological tools implemented for evaluation, and the methodology used for measuring CBF. In summary, it is not possible to precisely compare studies with different population subsets and nonreplicable methods.

Globally, regional CBF maps depicted by ASL may show both hypoperfusion or hyperperfusion areas at a global or localized scale, located in brain structures involved in different cognitive functions (Table 1).

More in detail, patients suffering from MCI depict areas of hypoperfusion in bilateral parietal lobes [7], posterior cingulate cortex (PCC) and precuneus [10, 13], left (L) occipital lobe, and bilateral frontal and temporal lobes [14], compared to healthy controls (HC). Interestingly, one study also found an increase in perfusion in the L hippocampus, right (R) amygdala, and basal ganglia (including rostral head of the R caudate nucleus, ventral putamen, and globus pallidus) [10].

Considering patients with fully developed AD, hypoperfusion areas are also present in the PCC and precuneus, bilateral parietal and temporal lobes, and bilateral superior and middle frontal gyri [6–8, 10, 12–14]. Additionally, temporooccipital and parietooccipital association cortices [5], L limbic lobe [14], and L orbitofrontal cortex [10] also depicted a hypoperfusion in certain studies [5–7, 9, 10, 12–15, 28]. Similarly to MCI, an increase in perfusion has been shown in the hippocampus [9, 11] and basal ganglia (putamen, caudate, lentiform, and thalamus) [14]. Additionally, anterior cingulate cortex (ACC) [10] and R limbic lobe [14] have also demonstrated a hyperperfusion in AD patients. Conversely, Asllani et al. [8] and Bron et al. [15] reported a hypoperfusion in medial temporal lobe structures [8, 15]. All abovementioned studies correspond to cross-sectional analysis comparing a pathological condition with a cognitively healthy state. Additionally, there is one longitudinal study including two groups of healthy patients at baseline, one of those exhibiting cognitive decline in follow-up visits. The “converter” group depicted a hypoperfusion in the PCC already at baseline [16], demonstrating that ASL might be a useful tool for predicting conversion to disease in the healthy elderly.

Additionally, nonsymptomatic high-risk groups may depict a hyperperfusion in the hippocampus, as individuals with a parental history of AD and at least one copy of APOE4 [11].

Finally, most of these studies have explored ASL in a resting state. Additionally, there are two studies using a memory-encoding task during the acquisition. When comparing task to baseline, patients with amnestic MCI [29] or at risk of AD (due to their APOE4 genotype and family history) [11] showed reduced activation in the parahippocampal gyrus [29] and hippocampus [11], compared to controls. It is relevant that these patients at risk of AD presented an elevated perfusion in the hippocampus at baseline [11], which might suggest that individuals with MCI or AD may lack the dynamic capability to modulate regional CBF in response to task demands.

From these studies we can extract that MCI and AD depict similar regions of perfusion changes, in particular more or less restricted areas of hypoperfusion in the PCC, precuneus, and other parietal regions, along with areas of hyperperfusion in the middle temporal lobe in some studies. In particular, the identification of a hypoperfusion in the PCC and precuneus had a sensitivity/specificity of 91%/80% [28], whereas using a combination of different regions in subjects with amnestic and dysexecutive MCI the accuracy was 60–70% [30].

Otherwise, the combination of structural and functional imaging in the same setting might contribute to the diagnosis of AD in clinical practice, as demonstrated in a study in which T1 weighted structural scans had 56/70% sensitivity/specificity in the detection of mild AD, while adding qualitative analysis of ASL increased these percentages to 85/54% [4].

Overall, the most consistent finding thorough the literature is a hypoperfusion in the PCC, which has been detected in all stages of AD (pre-MCI, MCI, and fully developed AD). Taking into account this piece of information, this finding might be considered as a functional biomarker of the disease.

## 4. Physiological Interpretation

Considering these studies, different patterns of CBF might coexist or evolve with the progression of AD. In early stages of the disease, certain areas such as the prefrontal cortex [31] or medial temporal lobe structures would eventually have the capability for “compensating” the cognitive decline, depicting an elevated atrophy-corrected perfusion. This compensatory mechanism could be explained by a pathological elevation of neural activity, release of inflammatory molecules, and/or increased blood supply through vascular dilation or increased vascular density [9, 32]. Simultaneously, perfusion deficits might be also present from the very early preclinical phases of AD, persisting into the latest stages of the disease, demonstrating a progressive hypoperfusion with disease development, leading to serious consequences on brain structure, cognition, and function in advanced stages of the disease.

Regarding the physiological interpretation of the regional hypoperfusion consistently found in the PCC, precuneus and/or lateral parietal cortex, a debate exists as to whether this is a cause or consequence of the disease [33]. It is known that vascular factors such as ischemic stroke, atherosclerosis, hypertension, diabetes, and cardiac disease are repeatedly implicated in the risk of developing AD [34]. While AD and vascular dementia have traditionally been considered as distinct entities, there is growing evidence of an overlap between these categories [35]. Furthermore, perfusion measures in the parietooccipital region [5] and parietal cortex along with the precuneus/posterior cingulate [6] have been shown to correlate with dementia severity (measured with the Blessed Dementia Scale [5] and MMSE [6]). Hypoperfusion may lead to changes in cortical thickness, as obtained from structural MRI scans, in areas most vulnerable to aging (medial prefrontal and pericentral cortices) as well as in areas associated with amyloid-aggregation (e.g., occipitotemporal and basal temporal cortices), especially in APOE4 carriers [36]. Finally, evidence from aging and stroke studies suggests that chronic brain hypoperfusion leads to neurodegeneration and cognitive impairment [33].

A hyperperfusion in medial temporal lobe structures has also been found with less consistency. The hypothesis of an increase of neural activity in these regions has been supported by two clinical studies [37, 38]. Choline acetyltransferase (ChAT), the enzyme responsible for synthesis of acetylcholine, was decreased in the hippocampi of patients suffering from MCI, whereas this elevation was no longer present in mild AD cases, and severe AD patients had markedly depleted levels. Cholinergic system might be capable of compensatory responses during early stages of dementia, so memory impairment in MCI might be the result of a progressive loss of entorhinal input to hippocampus, rather than a loss in hippocampal neural activity.

Additionally, alterations in the blood brain barrier and brain vasculature are known to be involved in neurodegeneration and neuroinflammation processes in AD. It has been suggested that a dynamic cycle of angiogenic events contributes to A*β* accumulation and neuronal death [39], and simultaneously amyloid-*β* (A*β*) plaques and neurofibrillary tangles (NFT), the pathological lesions found in the mesial temporal and neocortex of AD brains [40], may also be angiogenic [41, 42]. In a postmortem study comparing AD with HC brain tissues, the hippocampus was the only brain region exhibiting both angiogenic vessels (demonstrated using integrin *α*v*β*3 immunoreactivity) and increased vascular density [32]. This is consistent with the studies performed in APP23 transgenic animal models of AD, where *β*3 positive vessels were increased in disease susceptible regions [43], and also with studies linking AD to inflammatory mediators (TNF*α*, IL1*β*, IL-6, and IL-8), all of them with proangiogenic properties [44, 45], and with in vitro studies showing that microvessels of AD brains release angiogenic mediators such as angiopoietin-2 and vascular endothelial growth factor (VEGF) [46]. Finally, animal models have also shown that cognitive decline is enhanced following endothelial cell activation, proliferation, and subsequent vascular remodeling [43].

In conclusion, AD seems to behave as a dynamic process mediated by aberrant angiogenesis and inflammation mechanisms acting in certain vulnerable areas, leading to further neurodegeneration and progressive cognitive decline.

## 5. What Can ASL Add to the Standard Brain Perfusion Imaging Techniques?

In AD, perfusion (CBF and cerebral blood volume (CBV)) has been measured using a number of different imaging modalities, including MRI, xenon enhanced X-ray computed tomography (XeCT), or Single Photon Emission Computed Tomography (SPECT). Otherwise, PET studies provided the first maps of activation patterns in the human brain with high spatial resolution, by measuring changes energy metabolism [47]. Since then, numerous imaging techniques have been developed and applied to evaluate brain function [27], using different tracers (diffusible or nondiffusible, endogenous or exogenous), with different technical requirements. Brain perfusion imaging techniques also differ in terms of the duration of data acquisition, processing, quantitative accuracy, brain coverage, and spatial resolution, giving rise to the strengths and weaknesses of each technique for detecting alterations in neurodegenerative disorders (Table 2). For the past two decades, the nuclear medicine techniques SPECT and FDG-PET have served as the mainstream imaging techniques for perfusion and metabolism studies in AD. SPECT, however, has a low spatial resolution (around 1 cm), whereas FDG-PET has a 5 mm resolution and a higher sensitivity in low perfusion areas [31]. These techniques, however, require the use of exogenous radioactive tracers and are more expensive than the more recently developed perfusion-weighted MRI (PW-MRI) technique. Currently, PW-MRI is the most widely available technique for evaluating brain perfusion, by means of the dynamic susceptibility contrast (DSC) approach, which detects the first passage of an intravascular contrast agent such as gadolinium chelate, and the ASL technique, which uses magnetically labeled arterial blood water as a diffusible endogenous tracer. These two approaches measure different aspects of the perfusion state of the tissue. The contrast agent techniques provide a robust measurement of CBV, whereas the ASL techniques measure CBF, with the advantage of not requiring contrast injection. Many researchers are now migrating towards the use of ASL, since it poses less risk for the patients than DSC and nuclear medicine techniques.

A variety of studies in both animal models and human subjects have demonstrated that regional CBF maps can be accurately depicted by ASL [48–50], and the results obtained with ASL are consistent with those obtained from PET studies [51], since CBF and metabolic consumption are markers of cerebral dysfunction [50].

In clinical studies of AD and MCI, metabolic imaging with FDG-PET [52, 53] and SPECT [54, 55] have highlighted loss of activity in temporal, parietal, and frontal association cortex, along with PCC and precuneus. Additionally, a study using a covariance ^15^O-PET pattern [56] acquired during rest showed increased flow in the insula, cuneus, pulvinar, lingual, fusiform, superior occipital, and parahippocampal gyri, whereas decreased flow was found in cingulate, inferior parietal lobe, middle and inferior frontal, and supramarginal and precentral gyri. These results are in agreement with the findings reported in ASL studies.

In AD, medial temporal lobe is characterized by severe atrophy, so the detection of metabolism changes or subtle flow disturbances are difficult to evaluate with nuclear medicine techniques [57–59], since PET or SPECT studies do not correct for atrophy. Thus, one important advantage of combining ASL with structural MRI is the possibility of quantifying perfusion changes per cc of tissue in the same setting, allowing structure-function correlations within a single modality. Additionally, PET studies are prone to underestimate flow in regions with high blood flow, whereas ASL is less affected by water permeability due to the timescales of imaging and tracers decay [60]. Finally, SPECT can also suffer from saturation effects in the uptake of the tracer [61], and it has a low spatial resolution.

## 6. What Can ASL Add to the Diagnosis Flow of Patients with AD in the Amyloid-PET Era?

CSF studies and amyloid-PET imaging are considered molecular biomarkers of the disease, demonstrating the presence of the pathogenic protein “in vivo.” Its detection, in one way or another, is becoming increasingly important for establishing the diagnosis of probable AD [62]. However, recent evidence shows “positive” amyloid-PET studies in cognitively healthy individuals; thus these findings should be interpreted with caution [63]. Furthermore, it involves significant costs beyond the reach of most centers. On the other hand, ASL should be considered as functional or indirect biomarker, such as FDG-PET, showing focal abnormalities in perfusion within specific brain areas due to an underlying neurodegenerative process disrupting the neurovascular coupling [24]. Thus, amyloid-PET and ASL provide different but complementary information, which ideally might be integrated together in the diagnosis process of patients with cognitive decline. However, amyloid-PET requires great economic investment, which cannot often be carried out.

## 7. Limitations of the ASL Technique in Patients with MCI/Dementia

Discrepancies observed in different studies might be attributable to a large number of factors. First of all, there are significant differences in patients selection criteria, demographics, inclusion of other dementia syndromes, severity of the disease, neuropsychological tests used, and so forth. That is, the first study showing a hyperperfusion in the hippocampus included AD patients with unspecified severity and a mean age of 75.6 ± 9.2 years [9], whereas Asllani et al. study involved mild AD subjects with a mean age of 70.7 ± 8.7 years and Bron et al. subjects with different dementia syndromes, AD and frontotemporal lobe dementia, in presenile stages [15]. Secondly, methodological approaches are far from being standardized yet. Small differences between the commercial implementation of software or hardware from major MRI vendors may lead to a larger effect on the reproducibility of ASL [64]. Also, the different ASL techniques (continuous, pulsed, and pseudocontinuous), the wide range of acquisition parameters, and the different methodologies for data processing have led to an overabundance of options that has been translated in a lack of uniformity in results. Regarding acquisition parameters, most studies have used single postlabeling delay (PLD), whereas currently multiple-PLD is strongly recommended for AD [65]. Whereas a large proportion apply whole brain analysis (with or without atrophy correction), some works applied regions of interest (ROI) analysis [8, 15].

Finally, all these studies were interpreted using visual analysis; thus the subjective component is also a potential bias. This might be overcome in the future through different techniques of CBF quantification [23]. As mentioned before, a consensus statement on recommended implementation has been recently published for helping the clinical community to adopt a standardized approach on this technique, encouraging the use of pseudocontinuous labeling, background suppression, a segmented three-dimensional readout without vascular crushing gradients, and calculation and presentation of both label/control difference images and cerebral blood flow in absolute units using a simplified model [25].

Other potential drawbacks of ASL are the relatively low SNR, a problem which can be amplified in the clinical population because of lack of cooperation, vascular disease, and artifacts. Otherwise, assumptions regarding tagging efficiency, delay time to imaging, and flow quantification are based on predominantly normal populations in the research setting, and their translation to disease states has not been rigorously tested. Additional investigations are needed to define the impact of heart rate and cardiac output variations on image quality as well as how factors such as anemia, arterial stenosis, neurofibrillary tangles, vascular amyloid deposits, and atrophy may affect well-accepted parameters such as the T1 of blood used to quantify CBF. Coincidentally, some of the regions with the slowest arrival are those that also show flow decreases in AD, such as parietal and frontal association cortex. In third place, it can be challenging to obtain a good image quality in key regions for dementia research, characterized by strong magnetic susceptibility gradients (such as the inferior temporal lobes, the orbitofrontal cortex, and even the anterior medial temporal lobes), due to the proximity of these regions to air-tissue or bone-tissue interfaces [3]. Spin echo based sequences are particularly nonsensitive to these nonuniform magnetic fields [9, 66]. Finally, because of obstacles related to licensing as well as analysis of the ASL data, it has seen little in the way of routine application in clinical populations [67]. For these reasons, large clinical cohorts are needed to define the ranges of tissue-specific perfusion, researchers should strive to employ identical parameters, and quantification methods should be implemented to avoid bias of individual visual interpretations.

## 8. Conclusions

Brain perfusion measurement is a powerful neuroimaging tool for characterizing functional brain changes that occur early in the course of AD. The detection of a hypoperfusion in the PCC and precuneus seems to be the most consistent finding through different stages of the disease (and could potentially been used as a functional biomarker of AD), whereas a hyperperfusion depicted by mesial temporal lobe structures is more controversial. Current findings may provide new insights into AD pathophysiology, since these areas of hypo or hyperperfusion are promising regions for an early detection of this devastating disorder. Additionally, the ability to obtain regional CBF maps using a noninvasive and widely available modality such as ASL should greatly enhance the utility of blood flow measures as a means of gaining further knowledge into the broad range of hemodynamically related changes taking place during the cognitive decline process in AD.

## Figures and Tables

**Table 1:** Review of the literature regarding ASL findings in MCI and AD.

Ref	Methods	Number of subjects/MMSE (mean and range, in parentheses, or standard deviation (±), if available)	Patterns of hyper/hypoperfusion
[5]	(i) 1.5 T MRI (ii) EPISTAR: echo-planar imaging and signal targeting with alternating radio frequency (iii) Inclusion criteria: Hachinski Ischemia score < 4	(i) AD: 11 (ii) HC: 8	AD: ↓ in temporooccipital and parietooccipital association cortex, compared to HC

[6]	(i) 1.5 T MRI (ii) EPI CASL (iii) Inclusion criteria: Hachinski Ischemia score < 2	(i) AD: 17 (ii) HC: 11	AD: ↓ parietal, temporal, occipital, precuneus/posterior cingulate, and prefrontal cortex, compared to HC

[7]	(i) 1.5 T MRI (ii) PASL (iii) Voxel by voxel analysis. Correction for atrophy in the AD group	(i) AD: 20/21.0 (17–26) (ii) MCI: 18/27.7 (24–30) (iii) HC: 23/29.4 (28–30)	(i) AD: ↓ in bilateral inferior parietal cortex, PCC, bilateral superior and middle frontal gyri, compared to HC (ii) MCI: ↓ in inferior R parietal lobe, compared to HC

[8]	(i) 1.5 T MRI (ii) SE-EPI CASL (iii) Covariance pattern regional analysis using absolute, unnormalized, measures of blood flow	(i) AD: 12/38.7 ± 11.1 (ii) HC: 20/53.5 ± 2.8	AD: global decrease in flow (mean 40%), compared to HC. regional analysis using covariance pattern: ↓ in PCC, parahippocampal gyrus and hippocampus

[9]	(i) 3 T MRI (ii) 3D FSE CASL (iii) Atrophy corrected whole brain analysis	(i) AD: 22/22.2 (ii) HC: 16/27.9	(i) AD: ↓ in bilateral precuneus, parietal association cortex and L inferior temporal lobe, compared to HC. (ii) AD: ↑ in hippocampus and other medial temporal structures, after correction for GM loss, compared to HC

[10]	(i) 1.5 T MRI (ii) 2D CASL (iii) PVC applied for each voxel of CBF, probabilistic segmentation maps (iv) Voxel-level rCBF was compared among groups by using an analysis of variance design; clusters of voxels with significant group differences were identified	(i) AD: 37/85.1 ± 9.4 (ii) MCI: 26/90.8 ± 8.9 (iii) HC: 41/95.0 ± 4.5	(i) MCI, AD: ↓ in PCC and medial precuneus, compared to HC (ii) MCI: ↑ in L hippocampus, R amygdala, and rostral head of the R caudate nucleus, ventral putamen and globus pallidus, compared to HC (iii) AD: ↓ in L inferior parietal, L lateral frontal, L superior temporal, and L orbitofrontal cortices, relative to CN and MCI (iv) AD: ↑ in ACC, compared to CN

[11]	(i) 3 T MRI (ii) pASL (iii) Region of interest (ROI) analysis in the hippocampus	(i) Subjects at risk of AD (positive family history, at least one copy of apoE*ε*4): 13 (ii) Non-risk controls: 10	At risk group: ↑ in hippocampus (25%) at baseline

[12]	(i) 3 T MRI (ii) Multidelay PASL (iii) Measures of CBF, arterial transit delay and arterial blood volume	(i) AD: 19/20.1 ± 4.3 (ii) HC: 22/29.4 ± 0.9	AD: ↓ in precuneus and PCC (in all the measured parameters)

[13]	(i) 3 T MRI (ii) 3D FSE PCASL (iii) Whole-brain quantitative CBF (iv) PVC of CBF maps	(i) AD: 71/20.6 ± 4.6 (ii) MCI: 35/27.6 ± 1.9 (iii) SMC: 73/28.6 ± 1.7	MCI, AD: ↓ in parietal regions, precuneus and PCC, compared to SMC

[14]	(i) 3 T MRI (ii) 3D FSE PCASL (iii) Whole-brain quantitative CBF	(i) AD: 24/16.0 ± 3.9 (ii) MCI: 17/25.5 ± 2.2 (iii) HC: 21/29.4 ± 1.0	(i) *MCI Compared to HC* ↓ in bilateral frontal lobes and R temporal subgyral regions ↓ in L occipital lobe, bilateral inferior temporal cortex, and R middle temporal cortex (ii) *AD Compared with MCI* ↑ in R limbic lobe and basal ganglia (putamen, caudate, lentiform, thalamus) ↓ in L medial frontal lobe, parietal cortex, R middle temporooccipital lobe, and, particularly, the L anterior cingulate gyrus (iii) *AD Compared to HC* ↓ in bilateral temporoparietooccipital cortices and L limbic lobe

[15]	(i) 3 T MRI (ii) 3D FSE PCASL (iii) Voxel-wise method and a ROI-wise approach using five ROI-sets in the GM	(i) Presenile dementia at early stages (AD and FTD): 32/26.6 ± 2.9 (i) HC: 29 ± 1	↓ in the amygdala (L > R), L ACC, R PCC, bilateral thalamus, postcentral gyrus (R > L), bilateral inferior frontal gyrus, putamen (R > L), L insula, bilateral medial frontal gyrus, L superior frontal gyrus, L caudate, L occipital gyrus, bilateral gyrus parahippocampalis, bilateral medial temporal gyrus

[16]	(i) 3 T MRI (ii) 3D GE PASL (iii) Prospective longitudinal study	(i) sCON: 75/28.9 ± 1.1 (ii) dCON: 73/28.4 ± 1.2 (iii) MCI: 65/26.5 ± 2.3	↓ in the PCC at baseline is found in healthy elderly patients who develop subsequent cognitive deterioration

Ref: Reference. MMSE: Mini-Mental State Examination score. PASL: Pulsed ASL. CASL: Continuous ASL. PCASL: pseudocontinuous ASL. PVC: Partial volume correction. AD: Alzheimer**'**s disease. MCI: Mild cognitive impairment. HC: Healthy control. SMC: Subjective Memory Complaints. FTD: Frontotemporal Dementia. sCON: Stable Cognitive Function. dCON: Deteriorating cognitive function. ↓: Hypoperfusion in ASL. ↑: Hyperperfusion in ASL. R: Right. L: Left. FSE: Fast Spin Echo. GE: Gradient echo. CBF: Cerebral Blood Flow. GM: Grey Matter. PCC: Posterior Cingulate Cortex. ACC: Anterior Cingulate Cortex. SVM: Support Vector Machine.

**Table 2:** Overview of the imaging techniques dedicated to brain hemodynamics (adapted from Wintermark et al. [27]).

	PET	SPECT	DSC	ASL	fMRI
Age range	Adults (and children for static exams)	Adults (and children)	Adults (and children)	Adults + children	Adults (and children)

Contrast material	^15^O_2_, C ^15^O_2_, H_2_^15^O	^99^Tc-HMPAO, ^99^Tc-ECD, ^133^Xe, ^123^I-IMP (diffusible)	Gadolinium chelate (nondiffusible)	None (endogenous contrast)	None

Radiation/study	0.5–2 mSv	3.5–12 mSv	None	None	None

Data acquisition	5–9 min	10–15 min	1 min	5–10 min	

Data processing	5–10 min	5 min	5 min	5 min	

Assessed parameters	CBV, CBF, rOEF, glucose metabolism	CBF	CBF, CBV, MTT, TTP, permeability map	CBF	BOLD signal

Quantitative accuracy	Yes	Yes for ^133^Xe and ^123^I-IMP	Not in daily practice	Yes	

Including for low perfused areas	Yes	Not applicable	Not applicable	Not below 10 mL/min/100 g	Yes

Reproducibility	5%	10%	10–15%	10%	

Spatial resolution	4–6 mm	1 cm	2 mm	2 mm	

Minimal time interval between 2 successive exams	10 min	10 min	25 min	0 min	

Applications in neurodegenerative disorders	Yes	Yes	No	Yes	Yes
